# Responses of soil microarthropod taxon (Hexapoda: Protura) to natural disturbances and management practices in forest-dominated subalpine lake catchment areas

**DOI:** 10.1038/s41598-020-62522-w

**Published:** 2020-03-27

**Authors:** Maria Sterzyńska, Julia Shrubovych, Karel Tajovský, Peter Čuchta, Josef Starý, Jiří Kaňa, Jerzy Smykla

**Affiliations:** 1 Museum and Institute of Zoology, Polish Academy of Sciences, Wilcza 64, 00-679 Warsaw, Poland; 2 Biology Centre of the Czech Academy of Sciences, Institute of Soil Biology, Na Sádkách 7, 370 05 České Budějovice, Czech Republic; 3 Institute of Systematics and Evolution of Animals, Polish Academy of Sciences, Sławkowska 17, 31-016 Krakow, Poland; 4 State Museum of Natural History, Ukrainian Academy of Sciences, Teatral’na 18, UA 79008 L’viv, Ukraine; 5 Biology Centre of the Czech Academy of Sciences, Institute of Hydrobiology, Na Sádkách 7, 370 05 České Budějovice, Czech Republic; 6 Institute of Nature Conservation, Polish Academy of Sciences, Mickiewicza 33, 31-120 Kraków, Poland

**Keywords:** Ecosystem ecology, Animal behaviour

## Abstract

Disturbances are intrinsic drivers of structure and function in ecosystems, hence predicting their effects in forest ecosystems is essential for forest conservation and/or management practices. Yet, knowledge regarding belowground impacts of disturbance events still remains little understood and can greatly vary by taxonomic and functional identity, disturbance type and local environmental conditions. To address this gap in knowledge, we conducted a survey of soil-dwelling Protura, across forests subjected to different disturbance regimes (i.e. windstorms, insect pest outbreaks and clear-cut logging). We expected that the soil proturan assemblages would differ among disturbance regimes. We also hypothesized that these differences would be driven primarily by variation in soil physicochemical properties thus the impacts of forest disturbances would be indirect and related to changes in food resources. To verify that sampling included two geographically distant subalpine glacial lake catchments that differed in underlying geology, each having four different types of forest disturbance, i.e. control, bark beetle outbreak (BB), windthrow + BB (wind + BB) and clear-cut. As expected, forest disturbance had negative effects on proturan diversity and abundance, with multiple disturbances having the greatest impacts. However, differences in edaphic factors constituted a stronger driver of variability in distribution and abundance of proturans assemblages. These results imply that soil biogeochemistry and resource availability can have much stronger effects on proturan assemblages than forest disturbances.

## Introduction

Disturbances, natural and anthropogenic, are a common and intrinsic element of temperate forest ecosystems^1^ and play an important role in forest dynamics^2^. Seidl *et al*.^3^ have documented that across Europe, forest disturbance regimes have intensified markedly due to considerable increases in damage from insect outbreaks, windstorms and/or wildfires. As a result, growing disturbance risk necessitates greater attention to the management of the forest ecosystem^4^. Disturbance events such as insect outbreaks, windstorms and forest management practices applied after natural disturbances can strongly modulate forest structure, functioning and biodiversity^5,6^, and affect the natural, beneficial roles played by forests. Despite this the responses of species and taxa that inhabit disturbed forest ecosystems remain insufficiently understood. This kind of knowledge is essential to recognize the mechanisms and effects of disturbance regimes.

Soil biota contribute considerably to overall forest biodiversity and ecosystem services^7^. This belowground diversity controls soil processes that are critical to the functioning of forest ecosystems, such as litter decomposition and nutrient cycling^8,9^. The soil-litter system is inhabited by soil invertebrate communities that vary in diversity and in the type of food resources they utilize^10^. Soil microarthropods, including springtails (Collembola), oribatid mites (Acari, Oribatida) and proturans (Protura), being secondary consumers, play an important role in the decomposer food web through top-down control of the microbial decomposer community (i.e., bacteria and fungi)^11^, and contribute to soil microstructure and humus formation^12^. On the other hand, changes in resource availability related to natural forest disturbances (i.e., input of dead organic matter and decay of plant litter)^13^ cause a bottom-up effect that alters soil microbial communities and indirectly soil microarthropods^14^. Thus, soil biodiversity is a good indicator of soil quality^15^, and maintenance of high biodiversity is considered an integral component of forest management practices^16^.

Soil fauna is very sensitive to environmental disturbances; however, responses can vary greatly depending on taxonomic and functional identity and disturbance type^17^. Previous research on the impacts of forest disturbances on soil microarthropods has focused on the effects of wildfires^18^, windstorms^19^ insect pest outbreaks^20^ and forest management practices^19^, and has largely been limited to springtails and oribatid mites but neglected for proturans. Protura are distinctive litter and soil-dwelling invertebrates that are found in almost any place where decaying organic matter is deposited^21^. This mycophagous class of microarthropods primarily occurs in the rhizosphere of trees and appears to depend on the presence of ectotrophic mycorrhizal fungi^22,23^ and the physical condition of trees^21,22^.

However, as indicated above, proturans although acknowledged as a good model for ecological studies^24,25^ and indicators of the soil environment change via mycorrhizae^25^ have generally been neglected in ecological studies, and therefore virtually nothing is known regarding the impacts of various natural disturbance events and the post-disturbance forest management practices on their assemblages.

**Figure 1:**
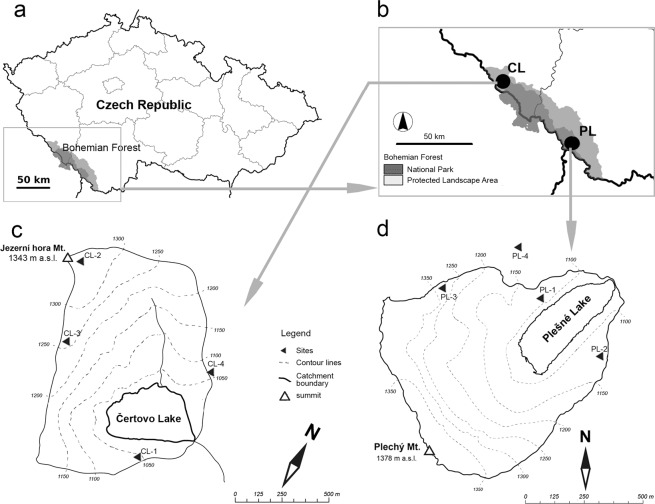
Map of the sampling sites in the Bohemian Forest National Park and Protected Landscape Area. CL – Čertovo Lake catchment, PL – Plešné Lake catchment: (**a**) location of the area studied in Czechia, (**b**) location of the examined catchments area at the Bohemian Forest, (**c**) study sites in Čertovo Lake (CL) catchment area, (**d**) study sites in Plešné Lake (PL) catchment area. Numbers refer to the study sites given in Table 1.

In this study, embedded in the extensive research programme “Effect of natural dieback of mountain spruce forests on microclimate, chemistry, and biodiversity of terrestrial and aquatic ecosystems” (e.g.^26^), we analysed proturan assemblages from the soils of two subalpine lake catchments in the Bohemian Forest, Czech Republic. This mountainous region comprises one of the largest stands of natural and semi-natural forests in Central Europe, and has a recent history of bark beetle outbreaks followed by and strong windstorms^13^. While some of these forests were left to regenerate naturally, others were subjected to active interventions with clear-cut logging. This provided a unique opportunity to investigate the responses of the proturan assemblages to different disturbance regimes and their cumulative impacts. Thus, a long-term ecological research program was developed and conducted in the area since 1984, with particular focus on two subalpine lake catchments – the Čertovo and Plešné Lakes (Fig. 1) – which differ in terms of underlying bedrock^27^.

It is widely recognized that geology plays an important role in determining the biodiversity and ecology of an area^28^. Beyond climate and topography, bedrock represents an important soil-forming factor which through the weathering process contributes to soil development and to modifications in local environmental conditions. The interaction between bedrock composition and surface processes regulates landscape evolution^29^. Accordingly, previous research conducted in the study area has demonstrated significant differences in soil biogeochemistry^27^ and soil microbial communities^30^ between the lake catchments. Consequently, differences in underlying bedrocks may affect the composition of proturan assemblages and their responses to the forest disturbances.

The main aim of this study was to assess the species composition and diversity of proturan assemblages in the subalpine lake catchments with different geological bedrock, as well as their responses to natural disturbance agents such as bark beetle outbreak and/or windthrow, and to post-disturbance forest management practices (clear-cut). We hypothesized that the differences in the soil physicochemical properties of the glacial lake catchments will affect species composition, diversity and abundance of proturan assemblages, whereas the impact of forest disturbances will be indirect and related to changes in food resources.

## Material and methods

### Study area

The study was conducted in the eastern part of the Bohemian Forest, known as the Czech Šumava Mts. (Fig. 1). The Bohemian Forest is a mid-sized mountain range with highest peaks exceeding 1400 m elevation. It extends ca. 100 km in a northwest-southeast direction across the Czech Republic, Germany (Bavaria) and Upper Austria. The mountain range shows the outcome of the effects of glacial activity, including the presence of glacial cirque lakes^31^, and is covered with vast, Norway spruce forests (*Picea abies* (L) Karst)^13^. Alongside the neighbouring Bavarian Forest, this mountain region comprises the largest forest complex in Central Europe. The original flora and fauna are relatively well-preserved, with the most valuable part protected within a transboundary mountain reserve that includes the Šumava National Park and Protected Landscape Area in the Czech Republic, and the Bavarian Forest National Park in Germany.

The area is characterized by a mixed continental and oceanic climate. The average annual temperature in the Šumava Mts. ranges between 6.0 °C (750 m elevation) and 3.0 °C (1300 m elevation). Mean monthly air temperatures vary from −5.3 °C in January to 12.3 °C in July. Annual precipitation varies between 800–1500 mm. Since the 1990s, a combination of mild winters, warm summers and storm events has favored the development of large populations of bark beetles (*Ips typographus*) in both the Czech and Bavarian parts of the Bohemian Forest, resulting in a large-scale dieback of Norway spruce^13^. In addition, in January 2007 the windstorm “Kyrill” damaged ca. 700,000 m^3^ of the spruce growth across 38.5% of the area^32^.

### Sampling sites

Sampling sites were established in two different catchment areas of the Čertovo (CL) and the Plešné (PL) Lakes, (Fig. 1). These catchments were located 67 km apart at 49°10′N, 13°11′E, and 48°47′N, 13°52′E, at elevations of 1030 m and 1090 m in the massif of Jezerní Hora Mt. (1343 m), and Plechý Mt. (1378 m), respectively. Both catchments are steep with the maximum differences in elevation reaching 313 m (CL) and 288 m (PL). The CL catchment covers an area of 89 ha (including the lake area of 10.7 ha) and the PL catchment covers 67 ha (with the lake area of 7.6 ha)^33^. The bedrock in the catchments is predominantly composed of mica-schist (muscovite gneiss), with quartzite intrusions in the CL catchment and granites in the PL catchment. Cambisols and Haplic Podzols predominate at both catchments^34,35^. The soil cover in the CL catchment is dominated (ca. 58%) by Cambisols, (0.49 ± 0.20 m deep), whereas in the PL catchment undeveloped, thin, organic-rich soils (O and A horizons, 0.20 ± 0.13 m deep) that are found across ca. 38% of the area are most frequent^34,35^.

Four spruce forest stands with different disturbance regimes were selected in both lake catchments including: 1) undamaged forests of 100–160 years old – control (abbreviations of stands: PL-1, CL-1); 2) forests damaged by bark beetles – BB (PL-2, CL-2); 3) windthrow forests subjected to bark beetle outbreak – wind+BB (PL-3, CL-3); 4) freshly harvested windthrow stands – clear cuts (PL-4, CL-4). The geographic coordinates and main features of the sampling sites are provided in Table 1.

**Table 1:** Characteristics and location of the sampling sites at the Plešné Lake (PL) and Čertovo Lake (CL) catchments in the Bohemian Forest.

Catchment	Plešné Lake (PL)				Čertovo Lake (CL)			
Bedrock	Granite				Gneiss			
Sampling site	PL-1	PL-2	PL-3	PL-4	CL-1	CL-2	CL-3	CL-4
Disturbance type	control	bark-beetle outbreak (BB)	wind+BB	clear-cut	control	bark-beetle outbreak (BB)	wind+BB	clear-cut
Elevation (m a.s.l)	1132	1123	1316	1001	1065	1339	1277	1047
Latitude N	48°46′39.6″	48°46′31.3″	48°46′34.7″	48°47′26.2″	49°09′46.4″	49°10′10.6″	49°09′57.8″	49°10′08.3″
Longitude E	13°51′51.5″	13°52′05.1″	13°51′23.3″	13°51′30.3″	13°11′58.5″	13°11′10.5″	13°11′17.1″	13°12′00.7″
Soil type	podzol	spodo-dystric cambisol	podzol	podzol	podzol	podzol	podzol	podzol
Soil horizon	O,A,E,Bh,Bs	O,A,E,Bh,Bs	O,A,Ae,C	ND	O,A,E,B,C	A,E,Bh,Bs,C	O,A,E,B	ND
Forest association	*Calamagrostio villosae-Piceetum abietis* var. *avenellosum* Jirásek 1996	*Calamagrostio villosae-Piceetum abietis* Schüter 1966	*Calamagrostio villosae-Piceetum abietis* Schüter 1966	*Junco effusi-Calamagrostietum villosae* Sýkora 1983	*Calamagrostio villosae-Piceetum abietis* Schüter 1966	*Calamagrostio villosae-Piceetum typicum* var. *avenellosum* Jirásek 1996	*Junco effusi-Calamagrostietum villosae* Sýkora 1983	*Junco effusi-Calamagrostietum villosae* Sýkora 1983
T_air_ (°C)	4.1	3.8	2.8	4.3	4.5	2.7	3.4	4.4

Soil types and soil horizons following Kopáček *et al*.^34,35^. ND – not determined. Plant (forest) associations and mean annual air temperature (T_air_) following Matějka^64^.

### Proturan sampling and laboratory analyses

Soil samples were collected twice a year for three consecutive years, i.e., in July and October 2012, June and October 2013, and June and September 2014. During each sampling session, five replicate soil cores were taken randomly with a steel corer. Each sample represented a soil core 3.6 cm in diameter (10 cm^2^ in area each), to a maximum depth of 7–12 cm (depending on the soil). The soil microarthropods were subsequently extracted in a modified high-gradient apparatus in the laboratory for seven days. All specimens of proturans were separated from extracted material under a dissecting microscope, fixed in ethanol and mounted in permanent microscope slides. Species identification was based on recent taxonomic keys^36–39^. All specimens were deposited in the collection of Institute of Systematics and Evolution of Animals PAS (Kraków, Poland). Abundance of proturan assemblages per each site was expressed as the density (D) of total number of specimens per m^2^. Subsequently, a set of assemblage parameters was calculated per each sampling occasion and site including, species richness (S) and Shannon’s diversity index (H’).

### Soil property analyses

To relate the distribution of proturan species to geochemical characteristics soils samples were collected from each of the sampling sites. These samples were collected once during the first sampling events (in July 2012) from the upper soil horizon (O; Ol+Of; up to 10 cm depth) in six pits (15 × 15 cm, 225 cm^2^). Prior to the analysis samples were stored at 4 °C in the dark for 3–5 days.

In the lab each soil sample was passed through a 5-mm stainless-steel sieve to remove coarse particles, and was then divided into two parts. One was analyzed for concentrations of water-extractable dissolved organic carbon (DOC), dissolved nitrogen (DN), and dissolved phosphorus (TP_H2O_) (water extract 1:10 by weight; field moist soil; 1 hour shaking on a horizontal shaker; filtration through glass fibre filters). Concentrations of DOC and DN were determined with a Formacs TOC/TN analyser (Skalar, Netherlands), and TP_H2O_ was determined by perchloric acid digestion and the molybdate method^40^. The pH was measured in distilled water (pH_H2O_) from naturally moist soils (1:10).

The other part of the soil sample was air-dried between two sheets of filter paper for 14–21 days at laboratory temperature, sieved through a stainless steel 2-mm sieve, then pooled to form a composite sample for each disturbance type per catchments and analyzed for exchangeable cations (CEC) and exchangeable acidity (Al^3+^_ex_ and H^+^_ex_). Exchangeable base cations (BC_ex_ = sum of Ca^2+^, Mg^2+^, Na^+^, K^+^) and exchangeable acidity (the sum of Al^3+^_ex_ and H^+^_ex_) were determined at natural soil pH by extracting 2.5 g of the soil with 50 ml of 1 *M* NH_4_Cl and 1 *M* KCl, respectively. Base cation concentrations were measured via atomic absorption spectrometry (Varian, Australia), and Al^3+^_ex_ and H^+^_ex_ were determined by titration (phenolphthalein, 0.1 *M* NaOH) according to Thomas^41^. Effective cation exchange capacity (CEC) was calculated as the sum of BC_ex_, Al^3+^_ex_ and H^+^_ex_. Base saturation (BS) was calculated as the percentage of BC_ex_ in CEC. The soil contents of DOC, DN and TP_H2O_ were based on three replicates, while CEC, BS, and Al^3+^_ex_ and H^+^_ex_ were based on the analysis of one mixed sample.

Soil moisture was measured during each sampling session weighting the soil cores collected for proturan analysis before and after extraction of animals. Gravimetrically determined soil moisture was expressed as the percentage of actual water content per soil dry weight. Soil temperature was measured with dataloggers (TidbiT v2, UTBI, Onset Computer Corporation, USA) throughout the entire research period (2012–2014). In order to calculate the mean soil temperature (T_soil_ °C) for individual study sites, the mean daily temperatures obtained for all sampling days were used.

To evaluate the relationship between variability of proturan assemblages and availability of their food resources the following approaches were undertaken: (1) dissolved organic carbon (DOC) concentration represented the main source of energy rich C substrates in soil for microbial decomposition^42^ and was considered as a proxy of microbial biomass and activity^42^; (2) dissolved nitrogen (DN) and total dissolved phosphorous (TP_H2O_) were considered as microbial nutrient limitation and used as a proxy of root colonization by mycorrhizal fungi^43^.

### Data analysis

The Kruskal-Wallis one-way ANOVA was used to compare differences in soil properties among sampling sites, representing different disturbance regimes. Furthermore, differences in soil properties between catchments were examined using the two-tailed Mann-Whitney U-test. A principal component analysis (PCA) was then performed to test whether variations in centered and standardized (z-scores) of the soil characteristics were able to discriminate the forest stands. In order to determine relevance of differences between the bedrock type, the PCA scores of weights of soil characteristics for the first and second axis were used as a synthetic dependent variable in the Mann-Whitney U-test.

Linear Mixed Model (LMM, Gaussian error, restricted maximum likelihood) was performed to analyze effect of disturbances regimes on value of Shannon diversity index (H’). Normal distribution of residuals was assumed. Forest disturbance regimes were used as a fixed factor and the nested time was used as a random factor (1 | year/season). The LMM model was run using the *lmer* function in the *nlme* R-package^44^ and *Imer Test* R-package^45^. Count data, such as Protura species richness (S) and density (D; expressed as thousands of individuals per m^2^) were analyzed using Generalized Linear Mixed Models. GLMM with Poisson error and maximum likelihood was applied to analyze the effects of disturbance regime on Protura species richness (S). Density of Protura (D) was analyzed using GLMM model with negative binomial error and maximum likelihood estimator. Model with negative binomial error was chosen because proturan density data were overdispersed. GLMMs were performed using *glmer* function of the Ime4 package^45^. LMM and GLMM models were fit with 3 groups of disturbances contrasted to undisturbed (control) sites. Manual model selection on the three possible models (using as random factor: season, year or the nested year/season) was carried out by the bias-corrected Akaike information criterion (AICc).

The effect of lake catchment, time and forest site disturbances on the variation in Protura assemblages composition was assessed with Canonical Correspondence Analysis (CCA). CCA was appropriate because the response data were compositional and had gradient length longer than 4.0 SD as determined by Detrended Correspondence Analysis (DCA)^46^. CCA was constrained by factors such as bedrock type (granite, gneiss), time (year, season), and forest stand disturbance regimes (control, bark beetle outbreak, windthrow and clear-cut). We also used partial CCA (pCCA) with time as covariate to corroborate differences in the composition of proturan assemblages between catchments and forest disturbance regimes. Decomposition of variation based on partial CCA ordination^47,48^ was performed to explain the unique effect (conditional) of the lake catchment (C), time (T) and forest disturbance regimes (FS) and the joint overlap of the investigated factors upon proturan assemblages. The adjusted variation using the number of degrees of freedom was applied. The significance of the models was estimated by the unconstrained Monte Carlo permutation test. We performed the multivariate analysis using the data matrix *species* × *forest stands* with the row data (number of individuals summed from five soil cores) collected at each of the forest stands during the same sampling session and then standardized to individuals per square meter. In the LMM, GLMM and multivariate analysis, time was treated as repeated measures for adjusting p-values as a function of correlation in the data. Before the multivariate analyses, proturan data were log(x + 1)-transformed to satisfy assumptions of normality and uniformity of variance. The level of significance in all analyses was at *α* = 0.05. Calculations were made using Statistica 10.0, Canoco 5.0^47^ software packages and R v. 3.4.4^49^.

## Results

### Edaphic factors

Descriptive statistics of the recorded edaphic properties for forest stands of the Plešné (PL) and Čertovo (CL) lake catchments are presented in Table 2. The two-tailed Mann-Whitney *U*- test demonstrated that the PL soils had significantly lower values of soil moisture (*U* = 0.0, z = −2.31, N = 4, p = 0.03) and dissolved nitrogen (DN) concentrations (*U* = 0.0, z = −2.31, N = 4, p = 0.03) but higher levels of dissolved phosphorus (TP_H2O_) (*U* = 0.50, z = −2.17, N = 4, p = 0.03) compare to the CL soils. However, no significant differences in mean ranks were recorded among the forest stands with various disturbance regimes for the dissolved organic carbon (DOC), dissolved nitrogen (DN), total dissolved phosphorus (TP_H2O_), total exchangeable capacity CEC, base saturation (BS), exchangeable aluminum (Al^3+^_ex_) and hydrogen (H^+^_ex_) (Kruskal-Wallis one way ANOVA:H (3, N = 8), *p* > 0.05 in all cases).

**Table 2:** Summary statistics (mean ± SD) of soil characteristics from the Plešné Lake (PL) and Čertovo Lake (CL) catchments in the Bohemian Forest.

Sampling site	PL-1	PL-2	PL-3	PL-4	CL-1	CL-2	CL-3	CL-4
Disturbance type	control	bark beetle outbreak (BB)	wind+BB	clear-cut	control	bark beetle outbreak (BB)	wind+BB	clear-cut
pH H_2_O	4.30	3.46	3.90	4.30	3.40	4.20	3.90	4.30
Moisture(%)	57.78 ± 13.92	78.46 ± 1.97	78.77 ± 2.25	69.40 ± 3.79	69.18 ± 5.67	74.26 ± 5.65	73.36 ± 5.47	72.40 ± 6.35
T_soil_ (°C)	7.4 ± 2.3	9.2 ± 2.5	8.2 ± 2.0	5.6 ± 2.0	7.6 ± 2.1	5.5 ± 1.3	8.2 ± 1.9	8.7 ± 1.1
DOC (mmol/kg)	50 ± 2.4	54 ± 15.5	59 ± 5.2	48 ± 1.4	43 ± 4.3	48 ± 3.0	43 ± 1.7	104 ± 2.6
DN (mmol/kg)	4.2 ± 0.6	8.8 ± 1.2	9 ± 0.4	12 ± 0.8	3.4 ± 1.1	4.8 ± 1.5	9.3 ± 0.4	16.8 ± 9.1
TP_H2O_ (mmol/kg)	0.15 ± 0.01	0.81 ± 0.07	0.98 ± 0.06	0.67 ± 0.02	0.14 ± 0.08	0.2 ± 0.02	0.7 ± 0.05	0.67 ± 0.03
CEC (meq/kg)	275	336	254	269	260	280	270	285
BS (%)	65.7	70	36	65.6	48	24.2	33.1	60.0
Al^3+^_ex_ (meq/kg)	26	21	76.7	27.4	41	104	98.6	50.7
H^+^_ex_	68	68	85	65	94	108	81	63

DOC – dissolved organic carbon, DN – dissolved nitrogen, TP_H2O_ – total dissolved P (in H_2_O extract), CEC – total exchangeable capacity [Mg^2+^ + Ca^2+^ + Na^+^ + K^+^ + Al^3+^_ex_ + H^+^_ex_], BS – base saturation [=(Mg^2+^ + Ca^2+^ + Na^+^ + K^+^)/(Mg^2+^ + Ca^2+^ + Na^+^ + K^+^ + Al^3+^_ex_ + H^+^_ex_) ×100%], and Al^3+^_ex_ and H^+^_ex_ – exchangeable aluminum and hydrogen.

**Figure 2:**
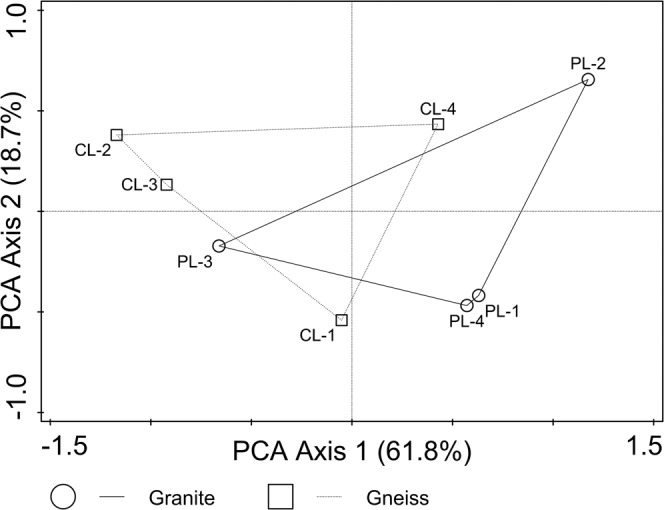
The PCA ordination biplot (PC 1 and PC 2) of the forest stands exposed to different disturbances, with subalpine lake catchments on various bedrock type) as the supplementary variable. The model calculated with row data of soil characteristics, interspecies correlation scaling, species score divided by SD and centring by species, not standardized by sample. Site abbreviations and numbering are given in Supplementary Table S1.

The results of PCA ordination corroborated the separation of soils on granite bedrock (PL sites) from those on mica-schist gneiss (CL sites), with significantly higher loadings for the first PC axis (two-tailed Mann-Whitney *U*-test for sum rang of soil properties: *p* = 0.0002; n = 8 in all cases), (Fig. 2). The PCA Axis 1 accounted for 61.8% of the total variance in soil properties and represented the negative correlations with exchangeable acidity (Al^3+^ and H^+^) and positive with base saturation (BS). The PCA Axis 2 appeared to be related positively to cation exchange capacity (CEC) and soil moisture, and accounted for 18.7% of the total variance (Supplementary Table S1). In the PCA plot (Fig. 2) the granite sites (PL) are on the right and the sites on gneiss (CL) on the left. Nonetheless, the PL-3 and CL-3 sites with bark beetle outbreak and windthrow (wind + BB) were located at a short distance on the diagram. These sites are characterized by a high content of acidic cations (Al^3+^, H^+^) and lower values of base saturation (BS).

### Species composition, abundance and diversity

A total of 146 specimens of Protura representing eleven species were recorded in the eight sampling sites in both lake catchments (Supplementary Table S2). The majority of the recorded proturans belonged to the genus *Eosentomon* Berlese, 1909 (10 species), while the genus *Acerentomon* Silvestri, 2007 was represented by a single species, *Acerentomon tuxeni* Nosek, 1961. The most widespread species was *Eosentomon gramineum* Szeptycki, 1986.

The generalized linear mixed model (GLMM) which contrasted disturbance regimes with undisturbed sites (control) demonstrated that combined disturbance regimes (wind + BB), besides disturbances by bark beetle outbreak (BB) and post-disturbance forest management practices (clear-cut), had significant and the strongest negative effect on Protura density (D). Furthermore, the evaluation of disturbance regimes on Protura species richness (S) using the generalized linear mixed model (GLMM), and on Shannon’s diversity index (H’) using the linear mixed model (LMM), also demonstrated negative impact of the combined disturbance regimes, such as bark beetle + windthrow. There was no statistically significant relationship between the value of Protura species richness (S) and disturbances by bark beetle outbreak (BB) or post-disturbance forest management practices (clear-cut) alone. However, in the case of H’ index, the lack of relationship was only found in response to bark beetle outbreak (BB) (Table 3). The variation in mean values of the basics parameters of Protura assemblages, such as density (D), species richness (S) and Shannon’s index of diversity (H’) among disturbance regimes in both catchments can be found as Supplementary Table S2.

**Table 3:** Summary of results of linear mixed model (LMM) including nested factor time as a random factor for Shannon index of diversity index (H’) and Generalized Linear Mixed Model (GLMM) for density (D 10^3^ ind. m^2^) and species richness (S) as a function of disturbance effect of damage by bark beetle (BB), bark beetle and windthrow (wind + BB) and clear-cut. Significant p-value is shown in bold.

	Estimate	SE	z(t)-value	p
**Density (D)**				
BB	−1.429	0.645	−2.214	**0.027**
wind+BB	−3.183	1.208	−2. 635	**0.008**
clear-cut	−1.486	0.656	−2.264	**0.024**
**Shannon diversity index (H’)**				
BB	−0.132	0.163	−0.812	0.422
wind+BB	−0.425	0.163	−2.613	**0.013**
clear-cut	−0.332	0.163	−2.042	**0.049**
**Species richness (S)**				
BB	−0.636	0.412	−1.543	0.123
wind+BB	−1.447	0.556	−2.604	**0.009**
clear-cut	−0.754	0.429	−1.758	0.079

**Figure 3:**
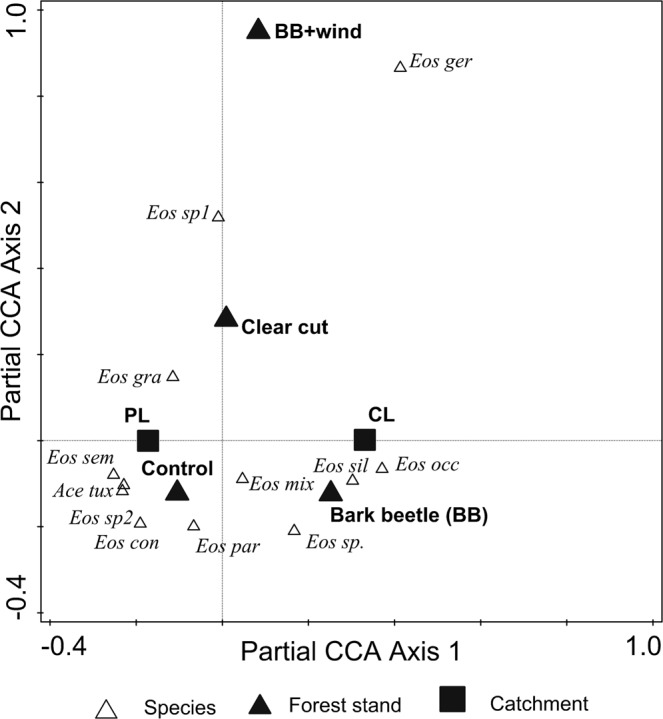
Distribution pattern of Protura species within upper mountain belt of forest. pCCA analysis with time (season and year) as covariate; model calculated with log(x + 1) transformed data. Species names and acronyms are given in Supplementary Table S2.

The total CCA analysis demonstrated that all of the investigated factors (i.e., lake catchment, time and forest stands) accounted for 43.1% of the variation (adjusted explained variation 12.8%) in the species dataset (λ-trace = 2.63, F-ratio = 1.42, p-value = 0.01). Variation decomposition (Table 4) demonstrated that lake catchment had the greatest unique effects within the model and accounted for 7.2% of the adjusted variation, whereas disturbance regimes and time were not significant and explained only a relatively small part of the variation (1.2% and 3.1%, respectively). Moreover, the joint overlap effect of all three factors was negative. The results of partial CCA (pCCA) with time as a covariate further corroborated differences in the composition of proturan assemblages between catchments and forest stands with different disturbance regimes showing significant species-environment relationships (λ-trace = 1.39, F-ratio = 1.50, p-value = 0.03). For instance, *Eosentomon silesiacum* Szeptycki, 1985 and *E. occidentale* Szeptycki, 1985 were associated with catchment sites underlying gneiss and *E. semiarmatum* Denis, 1927 and *Acerentomon tuxeni* Nosek, 1961 with sites underlying granite, whereas *E. germanicum* Prell, 1912 was associated with sites exposed to the effects of multiple disturbances due to both bark beetle outbreak and windthrow (wind + BB) (Fig. 3).

**Table 4:** Variance partitioning among catchments (C), time (T) and forest stands (FS) on variation in Protura assemblages from soils in the Bohemian Forest.

Model fraction	Explained variation (%)	Contribution to the total variation (%)	DF	Mean Square	pseudo-F	p-value
Unique C	56.8	7.2	1	0.540	2.3	**0.020**
Unique T	24.1	3.1	4	0.271	1.2	0.226
Unique FS	9.5	1.2	3	0.251	1.1	0.306
Overlap of C + T	5.8	0.7				
Overlap of C + FS	0.6	<0.1				
Overlap of FS + T	5.2	0.7				
Joint overlap of C + T + FS	−2.0	−0.3				
Total explained	100	12.7	8	0.330		
All variation	−	100	23			

Conditional effect performed by CCA and partial CCA model. DF– degree of freedom; mean square – denominator of F-statistic, pseudo-F – F-statistic; p – significance level of the effect tested by Monte Carlo permutation test, CCA model calculated with log(x + 1)-transformed data.Significant p-values are indicated in bold.

Variation account using the adjusted R^2^ approach.

## Discussion

One of the main goals of our study was to assess responses of proturan assemblages from montane forests to natural catastrophic disturbances and to post-disturbance forest management practices. To achieve that goal, our sampling design covered forests with four different type of disturbance, distributed across two subalpine glacial lake catchments representing variability in local geology. As expected, forest disturbance had negative effects on proturan diversity and abundance. However, results of the LMM and GLMM models (Table 3) demonstrated that multiple forest disturbance, i.e, bark beetle outbreak (BB) + windthrow significantly impacted proturan abundance, and value of Shannon’s diversity index and species richness. On the other hand differences in diversity index and richness were between undisturbed forests and sites affected only by bark beetle outbreak (BB) relatively smaller or statistically insignificant.

The apparent differences in proturan abundance, richness and Shannon’s diversity index related to a combination of natural disturbances (i.e., wind+BB) suggest that multiple disturbances can play an important role in shaping the response of proturan assemblages. This finding conforms to the concept that the effects of multiple disturbances can drive non-random and directional changes in the structure and diversity of belowground communities by provoking the decline of species, which is often explained by filtering effects of environmental changes on vulnerable traits^50^. Unfortunately it has been impossible to include in our sampling design sites with only windthrow disturbance, to discard the possibility that the combined BB + windthrow negative effects on proturans were due only or mainly to the latter factor. The results of the pCCA analysis (with time as a covariate) demonstrated that *Eosentomon germanicum* was the only species related primarily to sites on gneiss with a combined effects of natural disturbances (i.e., wind+BB). It is interesting to note, that *Eosentomon germanicum* is characterised by a wide ecological plasticity, and is broadly distributed in different habitats throughout Europe and Northern Africa^36^. This finding is in agreement with Coyle *et al*.^17^, who indicated that soil faunal responses to environmental disturbances are often species-specific and can be affected by complex environmental interactions. Furthermore, the withstand of forest disturbances by a widespread-generalist species of Protura shows that combined effect of natural forests disturbances can shift the specialist – generalist balance^51^. This finding also correspond well with Platt and Connell^52^ concept suggesting that directional replacement of species following natural disturbances favors “functional homogenization” of biodiversity^53^.

An important question pertaining to natural disturbances such as insect outbreaks and windthrow regards management intervention practices and their importance in mediating the impacts and restoration of forest ecosystems. Although the discussion relates primarily to the restoration of tree growth, understanding the effects of different forestry practices on belowground components, which mediate the supply of ecosystem services for aboveground components^54^, is also crucial in order to devise informed strategies of ecosystem management and conservation^17^. According to some studies^50^, forest management practices have no significant impact on soil-decomposer communities. Our study, however, highlights significant impact of post-disturbance clear-cut practices on diversity measures, richness and abundance of proturan assemblages. This is consistent with findings of Fischer *et al*.^1^, who demonstrated that management practices often have stronger impacts on forest ecosystems than the disturbances themselves. Post-disturbance clear-cuts have pronounced impacts on forest floor heterogeneity and considerably decrease natural buffering capacity of the soil by lowering organic matter contents^55^. Protura occur mostly in the tree rhizosphere; hence, the removal of trees from windthrow stands and subsequent environmental changes induced by soil erosion, loss of soil nutrients, modification of the soil moisture regime, increased sun exposure and changes in the soil microbial communities^56^ can all explain the negative implications for abundance and diversity of proturans in the soil.

Nonetheless, it is important to indicate that results of our study demonstrated that local differences in soil characteristics of subalpine glacial lake catchments, related to underlying bedrock, constituted more important driver of variability in distribution and abundance of proturan assemblages than forest disturbances. For instance, the variance partitioning showed that the catchment underlying various bedrock had the greatest unique effect on the variation in proturan assemblage composition and explained a more than twice larger part of the variation than the type of disturbance (Table 4). These high values support our hypothesis that differences in the physicochemical properties of soils, derived from different bedrock in the subalpine glacial lake catchments, will affect the species composition of proturan assemblages. This finding is consistent with Hahm *et al.*^29^ who pointed that bedrock composition can act as major bottom-up regulators of the montane ecosystems. But forest disturbances and tree dieback can also significantly influence the soil chemical properties and organic matter content across the study sites, and thus diversity and distribution patterns of the investigated proturan assembladges. The significant joint effect of both catchments and forest stands and negative shared variation implies that these variables overlap and that they have a additive effect on variations within the investigated proturan communities.

Taking into account enhanced values of total phosphorous and lowered of nitrogen content and moisture in soils developed on granite and their negative correlations with exchangeable acidity and positive with base saturation (BS) (i.e., Plešné Lake catchment, Table 2, Fig. 2, Supplementary Table S1) it is reasonable to expect that these variables provide the most important direct and/or indirect predictors explaining variation in proturan species distributions. Though, similarly to other soil microarthropods, differences in proturan assemblages are most likely associated also with differences in soil moisture and pH^22^, changes in the content of nutrients between these unsaturated, shallow and highly permeable catchment soils are linked via the soil microbial communities^57,58^. The same set of variables were usually linked to distribution, abundance and diversity in numerous studies of other soil microfauna e.g.^59^ indicating that the relationships found in our study between proturans and soil geochemistry could be generalized.

Protura, similarly to other soil microarthropods, participate in transformation and release of nutrients from organic matter, thus by affecting decomposition processes both directly and indirectly through comminution of litter, feeding on microorganisms and dispersal of microbial propagules^60^. Feeding on soil microbiota proturans comprise an important middle link in the soil food webs. According to Šantrůčková *et al*.^30^, the higher ability of granite to release phosphorous into the soil supports higher microbiologically mediated P-fluxes. This essential nutrient can directly mediate root colonization by mycorrhizal fungi^43^, which provide a major food resource for proturan^23^. Thus the relationship between proturans and differences in soil geochemistry between the catchments can be related to the indirect influence of bedrock on fungal community composition. However, it is important to highlight that geochemical factors can significantly influence distribution of soil invertebrates by affecting the availability of their food resources^56^. Although the lack of appropriate data on proturan feeding ecology and trophic relationships prevents us from informed connections between species distributions and their potential food resources, the preference of at least some species for ectotrophic mycorrhizal fungi was shown by some authors e.g.^23^. This indicates that proturans can be highly sensitive to soil geochemistry and environmental changes that impact mycorrhizae. For instance, an extraordinary high abundance of Protura was reported from a windfall area of a spruce forest three years after the storm^61^. These high densities were related to the amount and/or vitality of ectotrophic mycorrhiza associated with the fine roots of spruce seedlings in unsalvaged gaps. On the other hand, significant decline of proturans was recorded in a tree girdling experiment that constrained development of ectotrophic mycorrhizae fungi^62^. Considering the apparent importance of food resources, the high differences in proturan species abundances and their local distributions imply the existence of species-specific feeding preferences. In fact the dietary specialization can be more important driver of proturan distributions than the soil geochemistry and/or forest disturbances, which may act mostly as indirect drivers through their effects on ectomycorrhizal fungi and other decomposer rhizomorph producers^63^. Thus better knowledge of proturan feeding preferences might be the key to our understanding the importance of drivers underlying changes in distribution patterns of their assemblages. Focusing on this little known soil microarthropod taxon, our work provides a crucial baseline for further investigations on proturan diversity and understanding the impacts of environmental changes on distribution of their assemblages.

## Conclusions

To improve understanding of the impacts of forest disturbances on the diversity and distribution of soil arthropods, we investigated proturan assemblages in the glacial lake catchments of the Šumava Mountains in spruce forests subjected to different types of disturbances, including natural catastrophic events such as bark beetle outbreak and/or windthrow, as well as following forest management (clear-cut). As expected, forest disturbance had detrimental effects on proturan diversity and abundance, with multiple disturbances and clear-cuts having the greatest impacts. This result indicates that current management practices tend to have greater impact on the ecosystem than the natural catastrophic events themselves. Our findings also suggest that the composition of proturan assemblages was primarily driven by edaphic factors directly related to the type of bedrock. It is likely, however, that the observed associations between species distribution and soil geochemistry are a consequence of indirect relationships as geochemical factors can significantly influence distribution of secondary consumers by affecting the availability of their food resources.

## Supplementary information

Supplementary Information.
