# Supplementary material for: Responses of soil microarthropod taxon (Hexapoda: Protura) to natural disturbances and management practices in forest-dominated subalpine lake catchment areas
Source: Sci Rep. 2020 Mar 27;10:5572. doi: 10.1038/s41598-020-62522-w (PMC7101359; doi:10.1038/s41598-020-62522-w)
Supplement: Supplementary file 1 — Supplementary Information. [file 41598_2020_62522_MOESM1_ESM.docx]

Responses of soil microarthropod taxon (Hexapoda: Protura) to natural disturbances and management practices in forest-dominated subalpine lake catchment areas.

Maria Sterzyńska^1^, Julia Shrubovych^2,3,4^, Karel Tajovský^2*^, Peter Čuchta^2^, Josef Starý^2^, Jiří Kaňa^5^, Jerzy Smykla^6^

^1^Museum and Institute of Zoology, Polish Academy of Sciences, Wilcza 64, 00-679 Warsaw, Poland

^2^ Biology Centre of the Czech Academy of Sciences, Institute of Soil Biology, Na Sádkách 7, 370 05, České Budějovice, Czech Republic

^3^ Institute of Systematics and Evolution of Animals, Polish Academy of Sciences, Sławkowska 17, 31-016 Krakow, Poland

^4^ State Museum of Natural History, Ukrainian Academy of Sciences, Teatral’na 18, UA 79008 L’viv, Ukraine

^5^ Biology Centre of the Czech Academy of Sciences, Institute of Hydrobiology, Na Sádkách 7, 370 05, České Budějovice, Czech Republic

^6^ Institute of Nature Conservation, Polish Academy of Sciences, Mickiewicza 33, 31-120 Kraków, Poland

*Corresponding author.tajov@upb.cas.cz

Supplementary Table S1. Loadings of soil characteristics from PCA from forest stands at Plešné Lake (PL) and Čertovo Lake (CL) catchments in the Bohemian Forest.

| PCA component | Axis 1 | Axis 2 |
| --- | --- | --- |
| pH H_2_O | -0.09 | -0.07 |
| Moisture (%) | -0.28 | 0.60 |
| T_soil_ (ºC) | 0.32 | 0.39 |
| DOC (mmol/kg) | 0.25 | 0.42 |
| DN (mmol/kg) | 0.26 | 0.37 |
| TP_H2O_ (mmol/kg) | 0.09 | 0.35 |
| CEC | 0.58 | 0.74 |
| BS (%) | 0.99 | -0.09 |
| Al^3+^_ex_ (meq/kg) | -0.96 | 0.27 |
| H^+^_ex_ | -0.80 | -0.02 |

Supplementary Table S2**.** Species composition and the basic parameters of Protura assemblages of Protura assemblages in soils from different forest stands at the Plešné Lake (PL) and Čertovo Lake (CL) catchments in the Bohemian Forest. Abbreviations indicate: BB – bark beetle outbreak, wind+BB – windthrown and BB, clear-cut –freshly harvested windthrown stands. Values represent means ± standard deviation (SD) across repeated measurement of each site (n=5); D – density in 10^3^ ind. m^-2^.

| Catchment |  | Plešné Lake (PL) - granite | | | | Čertovo Lake (CL) - gneiss | | | | |
| --- | --- | --- | --- | --- | --- | --- | --- | --- | --- | --- |
| Disturbance type |  | control | BB | wind+BB | clear-cut | control | BB | wind+BB | clear-cut |  |
| Forest stand | acronim | PL-1 | PL-2 | PL-3 | PL-4 | CL-1 | CL-2 | CL-3 | CL-4 |  |
| *Acerentomon tuxeni* Nosek, 1961 | *Ace tux* | * | * |  |  |  |  |  |  |  |
| *Eosentomon* cf*. condei* | *Eos con* | * |  |  |  |  |  |  |  |  |
| *E. germanicum* Prell, 1912 | *Eos ger* |  |  |  |  |  |  | * |  |  |
| *E. gramineum* Szeptycki, 1986 | *Eos gra* | * | * | * | * | * |  | * |  |  |
| *E. mixtum* Condé, 1945 | *Eos mix* |  | * |  |  | * |  |  |  |  |
| *E. occidentale* Szeptycki, 1985 | *Eos occ* |  |  |  |  | * | * |  | * |  |
| *E. parvum* Szeptycki, 1985* | *Eos par* | * |  |  |  |  |  |  |  |  |
| *E. semiarmatum* Denis, 1927 | *Eos sem* | * |  |  |  |  |  |  |  |  |
| *E. silesiacum* Szeptycki, 1985 | *Eos sil* |  | * |  |  | * | * |  | * |  |
| *Eosentomon* sp*. bohemicum* gr*.* | *Eos sp1* |  |  | * | * |  |  |  |  |  |
| *Eosentomon* sp*. germanicum* gr*.* | *Eos sp2* | * |  |  |  |  |  |  |  |  |
| *Eosentomon* sp. juv*.* | *Eos sp.* |  | * |  |  |  |  |  |  |  |
| Density (D) |  | 3.24±4.04 | 0.32±0.30 | 0.08±0.10 | 0.80±1.14 | 0.76±0.74 | 0.64±0.99 | 0.08±0.10 | 0.10±0.09 |  |
| Species richness (S) |  | 2.20±1.60 | 1.00±0.89 | 0.40±0.49 | 0.80±0.75 | 1.20±0.98 | 0.80±0.75 | 0.40±0.49 | 0.80±0.75 |  |
| Shannon’s diversity index (H’) |  | 0.63±0.53 | 0.28±0.34 | - | 0.05±0.09 | 0.22±0.44 | 0.11±0.22 | - | 0.14±0.28 |  |

*Among the recorded species *Eosentomon parvum* Szeptycki, 1985 is a new species for Czech Republic
